# *N*′-(1-Phenyl­ethyl­idene)isonicotinohydrazide

**DOI:** 10.1107/S1600536809048569

**Published:** 2009-11-21

**Authors:** Jin-he Jiang, Jing Chen, Jie Yang, Fang-Fang Jian

**Affiliations:** a Microscale Science Institute, Weifang University, Weifang 261061, People’s Republic of China; b East China University of Science and Technology, School of Chemical Engineering, Shanghai 200237, People’s Republic of China

## Abstract

The title compound, C_14_H_13_N_3_O, was prepared from hypnone and isoniazid. The dihedral angle between the aromatic rings is 12.21 (2)°. In the crystal, N—H⋯O hydrogen bonds link the mol­ecules into chains propagating in [001] and C—H⋯O inter­actions consolidate the packing.

## Related literature

For background on Schiff bases, see: Cimerman *et al.* (1997 ▶). For a related structure, see: Chen *et al.* (2006 ▶).
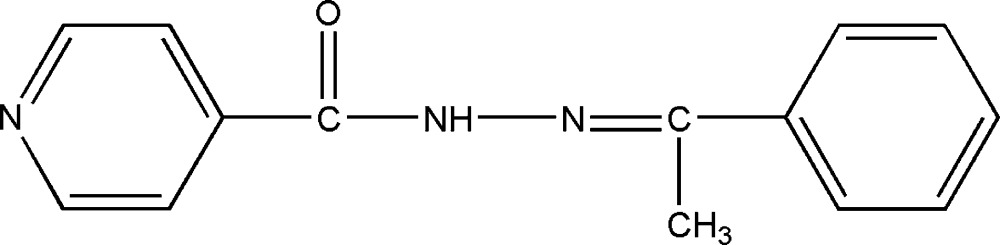


## Experimental

### 

#### Crystal data

C_14_H_13_N_3_O
*M* *_r_* = 239.27
Monoclinic, 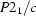

*a* = 25.878 (5) Å
*b* = 5.7100 (11) Å
*c* = 8.3089 (17) Å
β = 90.94 (3)°
*V* = 1227.6 (4) Å^3^
*Z* = 4
Mo *K*α radiation
μ = 0.09 mm^−1^
*T* = 293 K
0.35 × 0.25 × 0.25 mm

#### Data collection

Bruker SMART CCD diffractometer
Absorption correction: none
11394 measured reflections
2821 independent reflections
2024 reflections with *I* > 2σ(*I*)
*R*_int_ = 0.046

#### Refinement

*R*[*F* ^2^ > 2σ(*F* ^2^)] = 0.068
*wR*(*F* ^2^) = 0.205
*S* = 1.03
2821 reflections
167 parameters
H atoms treated by a mixture of independent and constrained refinement
Δρ_max_ = 0.24 e Å^−3^
Δρ_min_ = −0.29 e Å^−3^

### 

Data collection: *SMART* (Bruker, 1997 ▶); cell refinement: *SAINT* (Bruker, 1997 ▶); data reduction: *SAINT*; program(s) used to solve structure: *SHELXS97* (Sheldrick, 2008 ▶); program(s) used to refine structure: *SHELXL97* (Sheldrick, 2008 ▶); molecular graphics: *SHELXTL* (Sheldrick, 2008 ▶); software used to prepare material for publication: *SHELXTL*.

## Supplementary Material

Crystal structure: contains datablocks global, I. DOI: 10.1107/S1600536809048569/hb5224sup1.cif

Structure factors: contains datablocks I. DOI: 10.1107/S1600536809048569/hb5224Isup2.hkl

Additional supplementary materials:  crystallographic information; 3D view; checkCIF report

## Figures and Tables

**Table 1:** Hydrogen-bond geometry (Å, °)

*D*—H⋯*A*	*D*—H	H⋯*A*	*D*⋯*A*	*D*—H⋯*A*
N3—H3*A*⋯O1^i^	0.87 (2)	2.04 (3)	2.914 (2)	177 (2)
C4—H4*A*⋯O1^i^	0.93	2.43	3.123 (3)	131
C7—H7*A*⋯O1^i^	0.96	2.31	3.095 (3)	138

Symmetry code: (i) 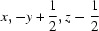.

**Fig. 1.**
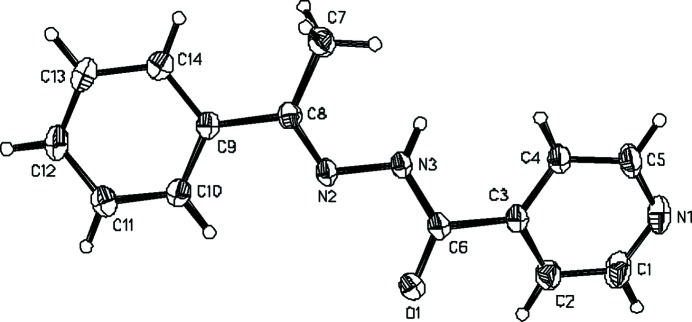
The molecular structure of (I) with displacement ellipsoids drawn at the 30% probability level.

## supplementary crystallographic information

### Comment

Schiff bases have received considerable attention in the literature. They are attractive from several points of view, such as the possibility of analytical application (Cimerman *et al.*, 1997). As part of our search for new schiff base compounds we synthesized the title compound(I) and report its crystal structure herein.

In the title compound (I) (Fig. 1), the C8=N2 bond, 1.285 (3) Å, and the C6—N3 bond, 1.351 (3) Å, are both longer than those in a related compound (Chen *et al.*, 2006). All other bond lengths are within normal ranges. The dihedral angle between the benzene and pyridine rings is 12.21 (2)°. The structure of (I) is stabilized by C—H···O,*N*—H···O and C—H···N hydrogen bonds (Table 1).

### Experimental

A mixture of the isoniazid (0.05 mol) and hypnone (0.05 mol) was stirred in refluxing ethanol(30 ml) for 2 h to afford the title compound (yield 78%). Colourless bars of (I) were obtained by recrystallization from ethanol at room temperature.

### Refinement

The N-bound H atom was located in a difference map and freely refined. The other H atoms were fixed geometrically and allowed to ride on their attached atoms, with C—H distances = 0.97 Å, and *U*_iso_(H) = 1.2–1.5*U*_eq_(C).

### Crystal data

**Table:**

C_14_H_13_N_3_O	*F*(000) = 504
*M**_r_* = 239.27	*D*_x_ = 1.295 Mg m^−^^3^
Monoclinic, *P*2_1_/*c*	Mo *K*α radiation, λ = 0.71073 Å
Hall symbol: -P 2ybc	Cell parameters from 2024 reflections
*a* = 25.878 (5) Å	θ = 3.2–27.5°
*b* = 5.7100 (11) Å	µ = 0.09 mm^−^^1^
*c* = 8.3089 (17) Å	*T* = 293 K
β = 90.94 (3)°	Bar, colourless
*V* = 1227.6 (4) Å^3^	0.35 × 0.25 × 0.25 mm
*Z* = 4	

### Data collection

**Table:**

Bruker SMART CCD diffractometer	2024 reflections with *I* > 2σ(*I*)
Radiation source: fine-focus sealed tube	*R*_int_ = 0.046
graphite	θ_max_ = 27.5°, θ_min_ = 3.2°
ω scans	*h* = −33→33
11394 measured reflections	*k* = −7→7
2821 independent reflections	*l* = −9→10

### Refinement

**Table:**

Refinement on *F*^2^	Primary atom site location: structure-invariant direct methods
Least-squares matrix: full	Secondary atom site location: difference Fourier map
*R*[*F*^2^ > 2σ(*F*^2^)] = 0.068	Hydrogen site location: inferred from neighbouring sites
*wR*(*F*^2^) = 0.205	H atoms treated by a mixture of independent and constrained refinement
*S* = 1.03	*w* = 1/[σ^2^(*F*_o_^2^) + (0.1014*P*)^2^ + 0.6215*P*] where *P* = (*F*_o_^2^ + 2*F*_c_^2^)/3
2821 reflections	(Δ/σ)_max_ < 0.001
167 parameters	Δρ_max_ = 0.24 e Å^−^^3^
0 restraints	Δρ_min_ = −0.29 e Å^−^^3^

### Special details

**Table:**

Geometry. All e.s.d.'s (except the e.s.d. in the dihedral angle between two l.s. planes) are estimated using the full covariance matrix. The cell e.s.d.'s are taken into account individually in the estimation of e.s.d.'s in distances, angles and torsion angles; correlations between e.s.d.'s in cell parameters are only used when they are defined by crystal symmetry. An approximate (isotropic) treatment of cell e.s.d.'s is used for estimating e.s.d.'s involving l.s. planes.
Refinement. Refinement of *F*^2^ against ALL reflections. The weighted *R*-factor *wR* and goodness of fit *S* are based on *F*^2^, conventional *R*-factors *R* are based on *F*, with *F* set to zero for negative *F*^2^. The threshold expression of *F*^2^ > σ(*F*^2^) is used only for calculating *R*-factors(gt) *etc*. and is not relevant to the choice of reflections for refinement. *R*-factors based on *F*^2^ are statistically about twice as large as those based on *F*, and *R*- factors based on ALL data will be even larger.

### Fractional atomic coordinates and isotropic or equivalent isotropic displacement parameters (Å^2^)

**Table:**

	*x*	*y*	*z*	*U*_iso_*/*U*_eq_	
N3	0.28050 (6)	0.2893 (3)	0.9742 (2)	0.0401 (4)	
N2	0.23151 (7)	0.3104 (3)	1.0394 (2)	0.0414 (5)	
C6	0.31164 (7)	0.1224 (4)	1.0382 (2)	0.0366 (5)	
O1	0.30052 (6)	0.0099 (3)	1.15887 (18)	0.0472 (4)	
C9	0.15153 (7)	0.4982 (4)	1.0663 (2)	0.0364 (5)	
C8	0.20515 (8)	0.4938 (4)	1.0019 (2)	0.0356 (5)	
C3	0.36258 (7)	0.0813 (4)	0.9583 (2)	0.0375 (5)	
C4	0.38721 (8)	0.2432 (4)	0.8623 (3)	0.0446 (5)	
H4A	0.3719	0.3870	0.8390	0.054*	
C7	0.22348 (9)	0.6943 (4)	0.9029 (3)	0.0492 (6)	
H7A	0.2583	0.6658	0.8704	0.074*	
H7B	0.2223	0.8356	0.9654	0.074*	
H7C	0.2016	0.7107	0.8092	0.074*	
C10	0.13477 (8)	0.3243 (4)	1.1704 (3)	0.0476 (6)	
H10A	0.1574	0.2058	1.2021	0.057*	
C13	0.06659 (9)	0.6712 (5)	1.0781 (3)	0.0591 (7)	
H13A	0.0437	0.7887	1.0468	0.071*	
C11	0.08489 (9)	0.3254 (5)	1.2275 (3)	0.0564 (7)	
H11A	0.0744	0.2092	1.2982	0.068*	
C14	0.11683 (9)	0.6724 (5)	1.0213 (3)	0.0501 (6)	
H14A	0.1273	0.7911	0.9524	0.060*	
C2	0.38778 (9)	−0.1288 (5)	0.9907 (3)	0.0526 (6)	
H2B	0.3728	−0.2414	1.0561	0.063*	
N1	0.45942 (8)	−0.0143 (5)	0.8287 (3)	0.0630 (7)	
C12	0.05063 (8)	0.4978 (5)	1.1801 (3)	0.0558 (7)	
H12A	0.0169	0.4965	1.2170	0.067*	
C5	0.43514 (9)	0.1864 (5)	0.8015 (3)	0.0564 (7)	
H5A	0.4514	0.2966	0.7373	0.068*	
C1	0.43566 (10)	−0.1669 (5)	0.9235 (4)	0.0658 (8)	
H1B	0.4523	−0.3078	0.9460	0.079*	
H3A	0.2875 (9)	0.349 (5)	0.881 (3)	0.045 (7)*	

### Atomic displacement parameters (Å^2^)

**Table:**

	*U*^11^	*U*^22^	*U*^33^	*U*^12^	*U*^13^	*U*^23^
N3	0.0333 (9)	0.0442 (10)	0.0431 (9)	0.0044 (7)	0.0129 (7)	0.0055 (8)
N2	0.0334 (9)	0.0454 (11)	0.0459 (9)	0.0043 (7)	0.0134 (7)	0.0031 (8)
C6	0.0323 (9)	0.0408 (11)	0.0368 (9)	−0.0011 (8)	0.0055 (8)	−0.0025 (8)
O1	0.0431 (8)	0.0558 (11)	0.0431 (8)	0.0037 (7)	0.0106 (7)	0.0078 (7)
C9	0.0321 (9)	0.0400 (11)	0.0372 (10)	0.0010 (8)	0.0026 (8)	−0.0047 (8)
C8	0.0348 (10)	0.0366 (11)	0.0355 (9)	0.0004 (8)	0.0044 (8)	−0.0050 (8)
C3	0.0308 (9)	0.0431 (12)	0.0387 (10)	−0.0001 (8)	0.0042 (8)	−0.0065 (9)
C4	0.0328 (10)	0.0499 (13)	0.0513 (12)	−0.0003 (9)	0.0073 (9)	0.0020 (10)
C7	0.0497 (13)	0.0392 (13)	0.0590 (13)	0.0002 (9)	0.0154 (11)	0.0030 (10)
C10	0.0359 (11)	0.0496 (14)	0.0573 (13)	0.0044 (9)	0.0049 (10)	0.0088 (11)
C13	0.0416 (12)	0.0639 (18)	0.0720 (17)	0.0195 (11)	0.0061 (12)	0.0028 (13)
C11	0.0399 (12)	0.0642 (17)	0.0654 (15)	−0.0024 (11)	0.0128 (11)	0.0112 (13)
C14	0.0454 (12)	0.0478 (14)	0.0572 (13)	0.0081 (10)	0.0071 (11)	0.0061 (11)
C2	0.0435 (12)	0.0449 (14)	0.0697 (15)	0.0046 (10)	0.0102 (11)	0.0025 (12)
N1	0.0366 (10)	0.0716 (17)	0.0813 (16)	0.0040 (10)	0.0147 (10)	−0.0137 (13)
C12	0.0310 (10)	0.0702 (19)	0.0662 (15)	0.0045 (10)	0.0087 (10)	−0.0048 (13)
C5	0.0349 (11)	0.0729 (19)	0.0619 (14)	−0.0045 (11)	0.0135 (10)	0.0007 (13)
C1	0.0445 (13)	0.0534 (16)	0.100 (2)	0.0129 (11)	0.0110 (14)	−0.0080 (15)

### Geometric parameters (Å, °)

**Table:**

N3—C6	1.351 (3)	C7—H7C	0.9600
N3—N2	1.392 (2)	C10—C11	1.382 (3)
N3—H3A	0.87 (2)	C10—H10A	0.9300
N2—C8	1.285 (3)	C13—C12	1.371 (4)
C6—O1	1.229 (2)	C13—C14	1.391 (3)
C6—C3	1.504 (3)	C13—H13A	0.9300
C9—C14	1.387 (3)	C11—C12	1.378 (4)
C9—C10	1.391 (3)	C11—H11A	0.9300
C9—C8	1.496 (3)	C14—H14A	0.9300
C8—C7	1.492 (3)	C2—C1	1.385 (3)
C3—C4	1.384 (3)	C2—H2B	0.9300
C3—C2	1.390 (3)	N1—C5	1.324 (4)
C4—C5	1.385 (3)	N1—C1	1.332 (4)
C4—H4A	0.9300	C12—H12A	0.9300
C7—H7A	0.9600	C5—H5A	0.9300
C7—H7B	0.9600	C1—H1B	0.9300
			
C6—N3—N2	116.69 (17)	C11—C10—C9	120.8 (2)
C6—N3—H3A	119.8 (16)	C11—C10—H10A	119.6
N2—N3—H3A	121.1 (16)	C9—C10—H10A	119.6
C8—N2—N3	117.33 (18)	C12—C13—C14	120.4 (2)
O1—C6—N3	122.91 (18)	C12—C13—H13A	119.8
O1—C6—C3	119.80 (19)	C14—C13—H13A	119.8
N3—C6—C3	117.27 (18)	C12—C11—C10	120.4 (2)
C14—C9—C10	118.20 (19)	C12—C11—H11A	119.8
C14—C9—C8	121.05 (19)	C10—C11—H11A	119.8
C10—C9—C8	120.74 (18)	C9—C14—C13	120.6 (2)
N2—C8—C7	125.95 (18)	C9—C14—H14A	119.7
N2—C8—C9	114.75 (18)	C13—C14—H14A	119.7
C7—C8—C9	119.30 (18)	C1—C2—C3	118.5 (2)
C4—C3—C2	117.99 (19)	C1—C2—H2B	120.7
C4—C3—C6	124.4 (2)	C3—C2—H2B	120.7
C2—C3—C6	117.5 (2)	C5—N1—C1	116.4 (2)
C3—C4—C5	118.5 (2)	C13—C12—C11	119.6 (2)
C3—C4—H4A	120.8	C13—C12—H12A	120.2
C5—C4—H4A	120.8	C11—C12—H12A	120.2
C8—C7—H7A	109.5	N1—C5—C4	124.5 (2)
C8—C7—H7B	109.5	N1—C5—H5A	117.8
H7A—C7—H7B	109.5	C4—C5—H5A	117.8
C8—C7—H7C	109.5	N1—C1—C2	124.1 (3)
H7A—C7—H7C	109.5	N1—C1—H1B	117.9
H7B—C7—H7C	109.5	C2—C1—H1B	117.9

### Hydrogen-bond geometry (Å, °)

**Table:**

*D*—H···*A*	*D*—H	H···*A*	*D*···*A*	*D*—H···*A*
N3—H3A···O1^i^	0.87 (2)	2.04 (3)	2.914 (2)	177 (2)
C4—H4A···O1^i^	0.93	2.43	3.123 (3)	131
C7—H7A···O1^i^	0.96	2.31	3.095 (3)	138

Symmetry codes: (i) *x*, −*y*+1/2, *z*−1/2.
